# Linking whole-body angular momentum and step placement during perturbed human walking

**DOI:** 10.1242/jeb.244760

**Published:** 2023-03-29

**Authors:** Jennifer K. Leestma, Pawel R. Golyski, Courtney R. Smith, Gregory S. Sawicki, Aaron J. Young

**Affiliations:** ^1^ George W. Woodruff School of Mechanical Engineering, Georgia Institute of Technology, Atlanta, GA 30332, USA; ^2^ Institute for Robotics and Intelligent Machines, Georgia Institute of Technology, Atlanta, GA 30332, USA; ^3^ Parker H. Petit Institute for Bioengineering and Biosciences, Georgia Institute of Technology, Atlanta, GA 30332, USA; ^4^ Wallace H. Coulter Department of Biomedical Engineering, Georgia Institute of Technology, Atlanta, GA 30332, USA; ^5^ School of Biological Sciences, Georgia Institute of Technology, Atlanta, GA 30332, USA

**Keywords:** Balance recovery, Whole-body angular momentum, Foot placement, Locomotion stability

## Abstract

Human locomotion is remarkably robust to environmental disturbances. Previous studies have thoroughly investigated how perturbations influence body dynamics and what recovery strategies are used to regain balance. Fewer studies have attempted to establish formal links between balance and the recovery strategies that are executed to regain stability. We hypothesized that there would be a strong relationship between the magnitude of imbalance and recovery strategy during perturbed walking. To test this hypothesis, we applied transient ground surface translations that varied in magnitude, direction and onset time while 11 healthy participants walked on a treadmill. We measured stability using integrated whole-body angular momentum (iWBAM) and recovery strategy using step placement. We found the strongest relationships between iWBAM and step placement in the frontal plane for earlier perturbation onset times in the perturbed step (*R*^2^=0.52, 0.50) and later perturbation onset times in the recovery step (*R*^2^=0.18, 0.25), while correlations were very weak in the sagittal plane (all *R*^2^≤0.13). These findings suggest that iWBAM influences step placement, particularly in the frontal plane, and that this influence is sensitive to perturbation onset time. Lastly, this investigation is accompanied by an open-source dataset to facilitate research on balance and recovery strategies in response to multifactorial ground surface perturbations, including 96 perturbation conditions spanning all combinations of three magnitudes, eight directions and four gait cycle onset times.

## Teaser

**Summary:** Investigation of human walking during perturbations varying in magnitude, direction and timing indicates a significant influence of all three variables on whole-body angular momentum and step placement as well as strong correlations between whole-body angular momentum and step placement in the frontal plane but not the sagittal plane.

## INTRODUCTION

Our dynamic and non-uniform world requires constant adjustments to maintain stable locomotion. Whether walking along a rocky path or mistakenly stepping off a street curb, humans demonstrate remarkable adaptability and agility as they move about their environment. By investigating how the balance of these individuals is affected by environmental perturbations and what strategies they employ to recover balance, we can better understand how healthy individuals achieve this robustness. This understanding could be used to design therapy strategies and assistive devices for populations with balance impairments or to inspire control strategies for bipedal robots.

The research community has developed a multitude of measures to quantify stability during gait across various contexts (Bruijn et al., 2013), which frequently couple step placement with center of mass (COM) mechanics (Hof et al., 2005; Hof, 2008). Within this collection of biomechanical stability measures, whole-body angular momentum (WBAM) provides an understanding of the overall rigid body dynamics of an individual, incorporating the momentum of each body segment about the COM, as opposed to capturing COM translations alone (Popovic et al., 2004; Herr and Popovic, 2008). Humans are shown to tightly regulate WBAM during locomotion, which has made it a useful tool in studying how balance deviates on different terrains (Silverman et al., 2012), during maneuvers (Nolasco et al., 2019) and in response to perturbations (Martelli et al., 2013; Liu et al., 2018). Additionally, WBAM has been shown to reflect worsened balance in populations with balance impairments and correlate with clinical balance measures, emphasizing its clinical relevance (Silverman and Neptune, 2011; Nott et al., 2014). Recently published work used integrated WBAM (iWBAM) to evaluate the body's rotation about the COM, providing useful information regarding the body's pitching motion over a set amount of time (Liu et al., 2018). This depiction of the body's position change could be useful in understanding the transient changes to an individual's stability following environmental perturbations. This may also provide a fairer evaluation of diversely perturbed locomotion by quantifying net change over time rather than capturing instantaneous WBAM changes, which are more aptly reflected by the range or peak of WBAM.

Because a change in WBAM is the integral of the net moment about the COM, it can be controlled by altering the center of pressure (COP), therefore modulating the lever arm of the ground reaction force (GRF) about the COM. Two key balance strategies that allow COP modulation to recover balance during locomotion are stepping strategy and ankle strategy (Horak and Nashner, 1986; Hof et al., 2010). Stepping strategy involves modulating the step placement location of the foot at heel strike, enabling large and abrupt changes to the COP relative to the COM, therefore increasing the GRF lever arm, which corrects for instability (Hof, 2008). However, there is often a delay between the onset of instability and the execution of this strategy, as it is only enabled at heel strike (Hof et al., 2010; Reimann et al., 2017). Ankle strategy involves modulating stance limb ankle torque to provide a small shift of the COP under the base of support; though this COP deviation is small, it can be applied throughout stance and is faster acting relative to stepping strategy (Hof et al., 2010). Though both strategies are useful in combatting instability, stepping strategy is often thought to be required to address large perturbations, when small COP changes using ankle strategy are not sufficient for balance recovery (Reimann et al., 2017). Thus, the modulation of step placement is expected to be one of the main drivers of balance recovery in unstable environments.

Despite the large body of work on both balance and recovery strategies, there is relatively little work investigating the relationship between the two. Simple models using COM mechanics have proven useful in predicting step placement, in both steady-state and perturbed walking (Bauby and Kuo, 2000; Wang and Srinivasan, 2014; Vlutters et al., 2016; Joshi and Srinivasan, 2019). However, these models have shown weaker correlations in the sagittal plane relative to the frontal plane, suggesting that step width may be more tightly regulated in response to changes in stability relative to step length (Vlutters et al., 2016; Wang and Srinivasan, 2014). Incorporating information on the body's overall pitching behavior using WBAM may improve predictive potential in comparison to more simplified balance metrics. iWBAM is a good candidate to investigate this, as it quantifies the net change of the body's pitching behavior over time. Further investigating the relationship between balance and recovery strategies would help us better understand how humans coordinate their response and correct for instability.

A variety of destabilizing scenarios could be used to probe the relationship between balance and recovery strategies. One common way to do this is to systematically apply environmental perturbations. Various methodologies exist for inducing instability, with common perturbation paradigms being COM pulls (Vlutters et al., 2016; Tan et al., 2020), walking surface translations (Kazanski et al., 2020), treadmill belt slips (Berger et al., 1984; Golyski et al., 2022) and swing foot obstacle collisions (Eveld et al., 2021). Across these methodologies, perturbation characteristics of interest have been magnitude, direction and onset timing (Afschrift et al., 2019; Golyski et al., 2022; Li and Huang, 2022; Vlutters et al., 2016). Though all of these variables individually have proven influential in affecting balance and recovery strategy (Golyski et al., 2022; Martelli et al., 2013; Vlutters et al., 2016), no study has systematically varied magnitude, direction and timing in tandem. This is a key contribution of this work, as it provides insight into the diverse landscape of possible destabilizing scenarios during bipedal locomotion and a comprehensive set of data to investigate hypotheses about both balance and recovery strategies.

The objectives of this research were to (1) introduce a novel multivariate perturbation paradigm and the associated open-source dataset, (2) evaluate how perturbation magnitude, direction and onset time affect stability and recovery strategy, and (3) investigate the relationship between stability and recovery strategy during perturbed locomotion. Our hypothesis was that frontal plane iWBAM will correlate with step width and sagittal plane iWBAM will correlate with step length during perturbed locomotion. To address our hypotheses, we perturbed individuals during walking with surface translations of various magnitudes, directions and onset timings, which simulated a variety of destabilizing environments. Stability was quantified as iWBAM over a step, while the subsequent recovery strategy was quantified as step placement changes relative to steady state.

## MATERIALS AND METHODS

### Experimental protocol

All participants provided informed consent before participating in the protocol, which was approved by the Georgia Institute of Technology Institutional Review Board. Eleven healthy participants (7 male and 4 female, mean±s.d. age 24.5±3.4 years, height 175.1±7.2 cm, leg length 91.3±5.1 cm, mass 73.3±11.0 kg) walked at 1.25 m s^−1^ on a split belt instrumented treadmill mounted on a 6 degree-of-freedom actuated platform [Computer-Aided Rehabilitation Environment (CAREN), Motek Medical, The Netherlands]. Each session began with a 6 min warm-up period in which the participant walked at 1.25 m s^−1^ on the treadmill; the first 5 min allowed the participant to acclimate to the treadmill (Zeni and Higginson, 2010) and the last minute was recorded to provide data that were not used in this study. The participant then entered the perturbation portion of the study.

**Fig. 1.**
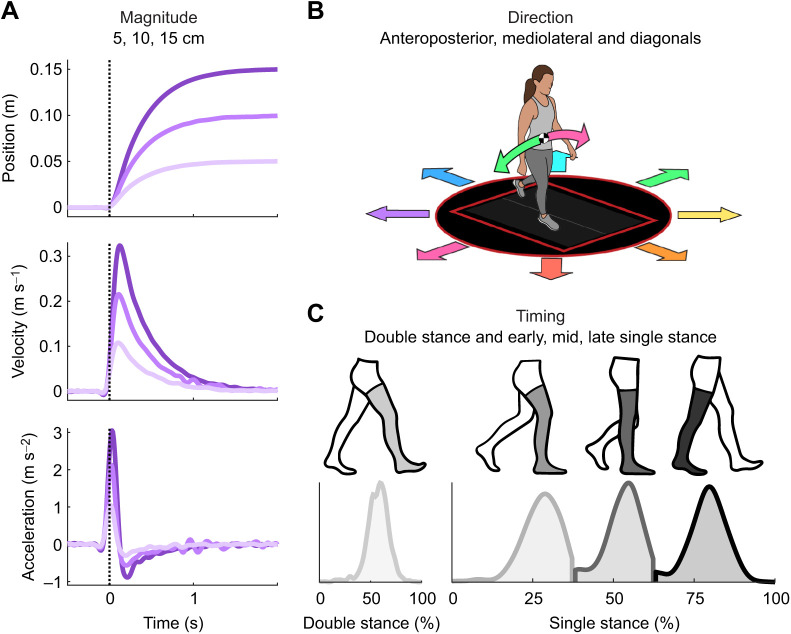
**Platform perturbation overview.** Perturbations varied in magnitude, direction and timing. (A) Magnitude included 5, 10 and 15 cm displacements of the ground underneath the participant. These overall displacement values were accompanied by changes in peak velocity and acceleration, as shown. (B) Direction included platform slides in the anteroposterior, mediolateral and corresponding diagonal directions. (C) Timing of the perturbation onset included double stance as well as early, mid and late single stance. Together, the complete combination of all these independent variables resulted in 96 unique conditions (3 magnitudes×8 directions×4 timings).

The participant walked while being exposed to intermittent perturbations where the walking platform translated in the transverse plane (i.e. ground height did not change, the platform slid along the plane defined by the global anteroposterior and mediolateral directions). Perturbations varied in three magnitudes (5, 10, 15 cm displacements), eight directions (anteroposterior, mediolateral and diagonals) and four targeted onset timings (50% double stance; 25%, 50%, 75% single stance) (Fig. 1). To control onset timing, we used real-time marker data (Vicon, Oxford, UK) and a kinematic gait event detection method (Zeni et al., 2008) to track single and double stance regions. We kept a running average of the duration of the prior three segment lengths of single and double stance phase for each leg, which we then used to command the perturbation at the desired onset time. The stance foot that the perturbation was initiated on was randomized to prevent participant anticipation of perturbation onset.

Each perturbation trial began with a randomized amount of time (∼4–10 s) before each perturbation to prevent participant anticipation of perturbation onset, as knowledge of perturbation timing has been shown to influence participants' responses (Major et al., 2020). The perturbation was then applied, and the platform remained statically at the perturbed position for 5 s to allow for return to steady-state walking before further platform movement. The platform then slowly returned to the center and the next trial began. Participants were instructed to gaze forward during the perturbation trials. The independent variables comprised 96 conditions, each of which was pseudo-randomly applied exactly once during a session. There were three perturbation sessions per participant that lasted approximately 40 min each, resulting in a total of 288 perturbations. Participants were given 5 min to rest between perturbation sessions.

### Data processing

We collected a full-body motion capture set containing 65 markers at 100 Hz (Vicon), including markers on the feet, shanks, thighs, pelvis, torso, head, upper arms, lower arms and hands. We also collected five markers on the treadmill platform. We collected analog data at 2000 Hz from two force plates embedded in the split-belt treadmill. Marker trajectories were lowpass filtered at 6 Hz using a second-order Butterworth filter. GRFs were lowpass filtered at 15 Hz using a fourth-order Butterworth filter.

We first calculated heel contact and toe-off events using a kinematic method (Zeni et al., 2008). We then determined the perturbation onset time when the platform displacement exceeded a 1 mm threshold. To determine the actual perturbation timing relative to the gait cycle, we repeated the method used to control the experiment *post hoc*: (1) we used a running average of single and double stance phases for each leg based on gait events, (2) determined the stance region (single or double) at the start of the perturbation, and (3) identified the percentage of that stance region at the start of the perturbation based on the running average duration of the stance region. Though perturbation onset timing primarily occurred in the targeted regions, some trials occurred during other target regions in the gait cycle. This likely occurred as a result of natural gait variability and mislabeling or gaps in the marker data used to track gait events in real-time. We *post hoc* binned the trials into the appropriate onset timing group; these groups included double stance and early (1–37.5%), mid (37.5–62.5%) and late (62.5–100%) single stance. This resulted in a small number of perturbation conditions being applied more or less than the intended three trials per participant; all perturbations were repeated at least once per participant, while the majority were repeated the desired three times.

### Calculation of WBAM

We calculated WBAM using OpenSim 4.1 (Delp et al., 2007; Seth et al., 2018). We used a full-body OpenSim model (Rajagopal et al., 2016) with expanded shoulder ranges of motion to enable better tracking of arm swing in response to perturbations. To this model, we added a custom marker set to match our experimental setup with additional virtual markers to aid in the scaling process. We scaled each generic musculoskeletal model using static marker trials and participant mass to generate models for each participant using the OpenSim Scale Tool with manually tuned marker weights. We analyzed the inverse kinematics of each trial using the OpenSim Inverse Kinematics Tool with manually tuned marker weights and custom Matlab scripts (Camargo et al., 2020) (Mathworks, Natick, MA, USA), which resulted in a mean root mean square (RMS) marker error of 2.49 mm and a mean peak marker error of 5.65 mm. We obtained the orientation and COM position for each of the 22 segments in the OpenSim model using the OpenSim Analyze Tool; this included the torso and pelvis as well as bilateral femur, patella, tibia, talus, calcaneus, toes, humerus, ulna, radius and hand segments. We then calculated WBAM according to:
(1)

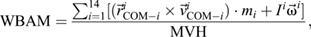
where 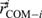 is the distance between the segment COM and the whole-body COM, 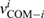 is the velocity of the segment COM relative to the whole-body COM, *m_i_* is the mass of the segment, *I^i^* is the inertia tensor of the segment and ω*^i^* is the angular velocity of the segment. Frontal and sagittal planes are defined in the global reference frame, with anteroposterior motion aligning with the direction of treadmill movement. WBAM was normalized by each participant's mass (*M*), walking velocity (*V*; 1.25 m s^−1^), and height (*H*). We calculated WBAM using custom Matlab scripts (Mathworks).

**Fig. 2.**
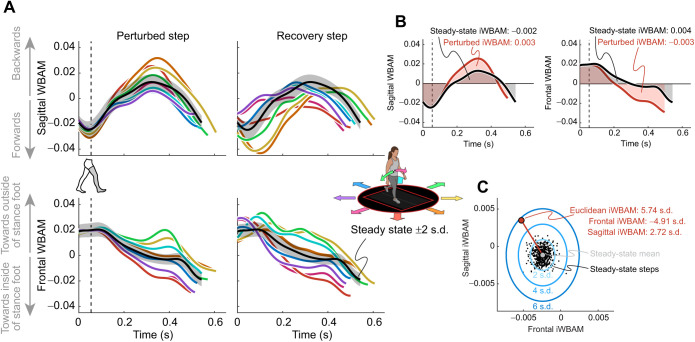
**Calculating integrated whole-body angular momentum during the perturbed and recovery steps.** (A) Whole-body angular momentum (WBAM) during steady-state and perturbed walking across directions for a representative participant. WBAM is normalized by participant mass, height and walking speed, resulting in a unitless value. The steady-state average and its ±2 s.d. are shown by the black line and gray region, respectively. Examples of perturbed strides are shown for each direction, with magnitude fixed at 15 cm and timing fixed at double stance. Approximate onset time is indicated by the vertical dashed line. Each direction is the average WBAM curve for a representative participant across all repetitions of the given condition. Directions are shown by different colors corresponding to the walking arrow diagram in the lower right panel. The center of mass (COM) pitches in the opposite direction to the platform slide. Generally, the green arrow results in a narrowing step while the pink arrow results in a widening step; the orange arrow pitches the COM backward, while the blue arrow pitches the COM forward. (B) Integrating sagittal and frontal WBAM. Integrated WBAM (iWBAM) is found by calculating the area under the WBAM curve with respect to time during a step. The perturbed steps (shown in red) are compared against the steady-state steps (shown in black). The shaded regions depict the integrated area under the curve. (C) Determining Euclidean iWBAM. The perturbed steps (example red point) are normalized relative to the steady-state steps (black dots). We first subtract the steady-state mean (light gray point) from the perturbed step and then divide by the standard deviation (2, 4, 6 s.d. shown by blue ovals) of the steady-state steps. This results in a value with units of s.d. and depicts how severely the perturbed and recovery steps deviate relative to steady-state data. Finally, we also calculate the Euclidean deviation of iWBAM (length of red line between mean and perturbed step) using the normalized frontal and sagittal iWBAM values.

The translational perturbations in this study used anteroposterior and mediolateral platform movements, which we anticipated would cause changes in sagittal and frontal WBAM, respectively. Because of this, we chose to analyze sagittal and frontal WBAM (Fig. 2A), though transverse WBAM is still included in the open-source dataset. We then used trapezoidal numerical integration to determine iWBAM in the frontal and sagittal plane over each step, defined by heel contact on one leg to heel contact on the contralateral leg. This was done for all steady-state steps that occurred in a trial prior to the onset of a perturbation, as well as for the perturbed step. To determine the recovery step (the step after the perturbed step) iWBAM value, we took the total integral over both the perturbed and recovery steps. Because iWBAM depicts the cumulative rotation of the body about the COM (i.e. pitch or roll; pitch will be used throughout this paper to depict multi-directional and/or net rotation) and we aimed to determine this total rotation at the conclusion of the recovery step, we integrated over both steps so the recovery step was not biased by the net rotation of the body at the end of the perturbed step. We then normalized all data to each participant's steady-state mean and standard deviation, which we used to quantify how much perturbations affected iWBAM and step placement relative to each participant's unperturbed walking. To do this, we (1) calculated the mean and standard deviation of frontal and sagittal iWBAM in the steady-state steps, (2) subtracted the steady-state mean from each perturbed data point, and (3) divided by the steady-state standard deviation. The resulting value has units of s.d. and quantifies an increase or decrease in iWBAM relative to steady state. We also used these results to calculate the Euclidean iWBAM, combining frontal and sagittal iWBAM to quantify overall deviation in iWBAM over a step, again with units of s.d. (Fig. 2B,C). Because previous work did not find a significant difference in WBAM in response to perturbations on the dominant and non-dominant legs (Martelli et al., 2013), all results for perturbations on the right and left legs were combined. Throughout this analysis, the perturbation direction is defined relative to the perturbed stance foot, where pink and green represent the platform moving laterally and medially relative to the perturbed foot, respectively. This normalization to the perturbed foot accounted for all perturbations with a mediolateral component, enabling the trials that perturbed the right and left feet to be combined. All figures and diagrams represent the perturbed foot using the right foot.

### Calculation of recovery strategies

**Fig. 3.**
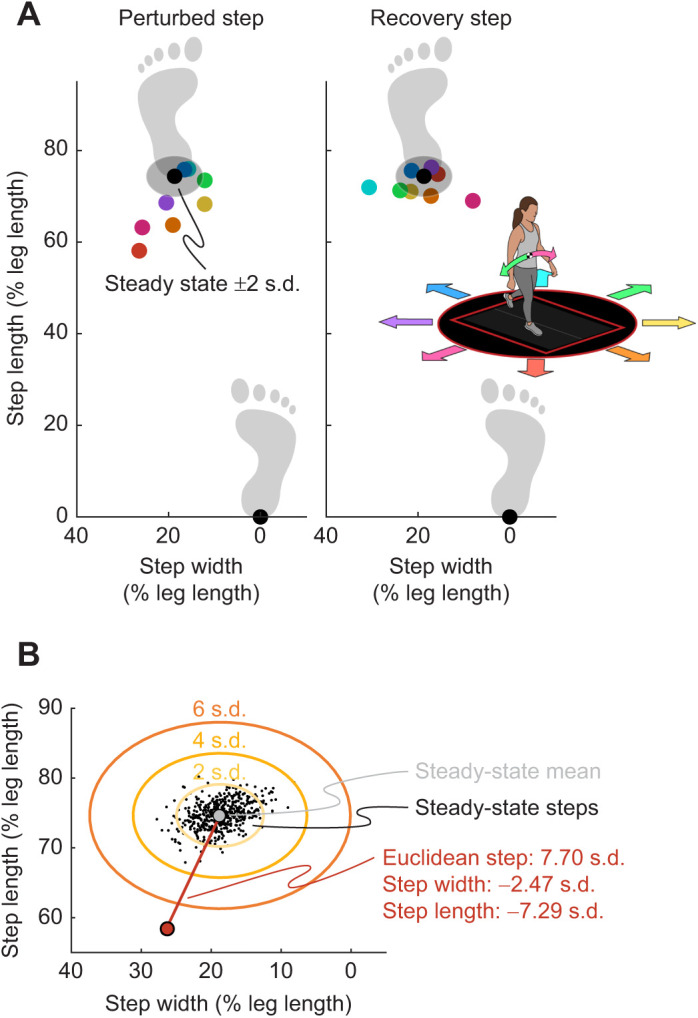
**Calculating step placement during the perturbed and recovery steps.** (A) Step placement during steady-state and perturbed walking across directions for a representative participant. Step placement is normalized by the participant's leg length. The steady-state average and ±2 s.d. are shown by the black point and surrounding shaded region, respectively. Examples of perturbed strides are shown for each direction, with magnitude fixed at 15 cm and timing fixed at early single stance. Each direction is the average step placement for a representative participant across all repetitions of the given condition. Directions are shown by colored points corresponding to the walking arrow diagram in the right panel. The COM pitches in the opposite direction to the platform slide. Generally, the green arrow results in a narrowing step while the pink arrow results in a widening step; the orange arrow pitches the COM backward, while the blue arrow pitches the COM forward. (B) Determining Euclidean step placement. The perturbed steps (example red point) are normalized relative to the steady-state steps (black dots). We first subtract the steady-state mean (light gray point) from the perturbed step and then divide by the standard deviation (2, 4, 6 s.d. shown by orange ovals) of the steady-state steps. This results in a value with units of s.d. and depicts how severely the perturbed and recovery steps deviate relative to steady-state data. Finally, we also calculate the Euclidean deviation of step placement (length of red line between mean and perturbed step) using the normalized step length and width values.

We calculated step length and width as the anteroposterior and mediolateral distance between heel markers, respectively, at each heel contact. Similar to iWBAM calculations, we then normalized all data to the steady-state mean and standard deviation to determine how significantly step placement deviated in the perturbed and recovery steps. The normalization procedure described for iWBAM was repeated, resulting in values with units of s.d. and quantifying an increase or decrease in step placement relative to steady state. We also used these results to calculate the Euclidean step placement, combining step width and length to quantify overall deviation in step placement during a step, again with units of s.d. (Fig. 3).

Several participants exhibited a jumping strategy in response to perturbations. In these cases, participants were in single stance and pushed off the ground, resulting in a period of no ground contact. To detect these cases, we evaluated the combined vertical GRFs between both force plates and identified jumps during periods where the combined force fell below 40 N. Because a comparable step length and width calculation could not be made for these cases, we treat this as a special case stepping strategy.

### Statistics

We first sought to investigate the effect of perturbation magnitude, direction and timing on iWBAM and step placement. We performed a three-way repeated measure analysis of variance (ANOVA) to determine the effects of perturbation magnitude, direction and timing, as well as their interaction effects, on Euclidean iWBAM and step placement during both the perturbed and recovery steps (Minitab 19). Significance was set at α=0.05.

To address our hypothesis, we evaluated the relationship between frontal iWBAM and step width as well as sagittal iWBAM and step length in both the perturbed and recovery steps for different perturbation onset times across all participants. We fitted each relationship with a linear best fit line and determined the significance (*P*-value) and coefficient of determination (*R*^2^) using custom Matlab scripts (Mathworks). We also evaluated the coefficient of determination (*R*^2^) for each correlation for individual participants, which was used to evaluate the effect of onset timing on the strength of the correlation. We performed a one-way repeated measure ANOVA to determine the effect of perturbation onset timing on the strength of correlations between iWBAM and step placement (Minitab 19).

## RESULTS

### Perturbation conditions and balance

**Fig. 4.**
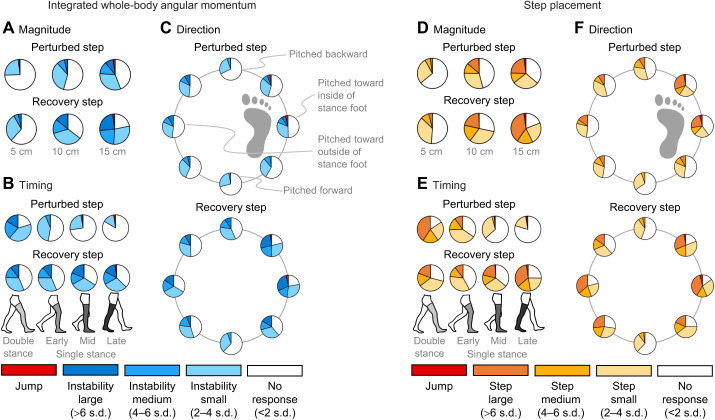
**iWBAM and step placement changes across perturbation conditions.** Results from all participants are shown. (A–C) Deviations in balance, measured by changes in iWBAM relative to steady state, across perturbation conditions for the perturbed and recovery steps (A, magnitude; B, timing; C, direction). Severity of deviation is classified as no response as well as small, medium and large changes in stability. An additional class, jump, is shown for trials in which the participant lost ground contact. If a jump occurred following the perturbation, the jump classification is shown for both the perturbed and recovery step. (D–F) Deviations in step placement across perturbation conditions for the perturbed and recovery steps (D, magnitude; E, timing; F, direction). Severity of deviation is classified as no response as well as small, medium and large changes in step placement. Again, jump is shown for trials in which the participant lost ground contact. If a jump occurred following the perturbation, the jump classification is shown for both the perturbed and recovery step.

We evaluated how changes in balance, measured by the deviation in Euclidean iWBAM from steady-state walking, were affected by the magnitude, direction and timing of a perturbation (Fig. 4A–C; Tables S1, S2 and S3). The ANOVA revealed that magnitude, direction and timing all significantly affected Euclidean iWBAM in the perturbed step (all *P*<0.001). Also, for the perturbed step, there was a significant interaction of magnitude/direction, magnitude/timing, direction/timing and magnitude/direction/timing (all *P*<0.001). The ANOVA also revealed that magnitude, direction and timing all significantly affected Euclidean iWBAM in the recovery step (all *P*<0.001). Also, for the recovery step, there was a significant interaction of magnitude/direction, direction/timing and magnitude/direction/timing (*P*<0.001), but not of magnitude/timing (*P*=0.125). In both the perturbed step and the recovery step, magnitude tended to increase balance deviation, with the lowest magnitude rarely causing more than a small response. Perturbations with onset timing later in the gait cycle caused less severe balance deviation in the perturbed step. However, balance deviations in the recovery step had more comparable distribution across all onset timings, with the most severe deviations occurring for the later onset times (mid and late single stance). For perturbation direction, in both the perturbed step and recovery step, anteroposterior perturbations caused the least severe balance deviation while perturbations with a mediolateral component tended to cause greater balance deviations. Fig. S1 is a comparable figure that individually evaluates frontal and sagittal iWBAM.

### Perturbation conditions and recovery strategies

We evaluated how changes in Euclidean step placement relative to steady-state walking were affected by the magnitude, direction and timing of a perturbation (Fig. 4D–F; Tables S1, S2 and S3). The ANOVA revealed that magnitude, direction and timing all significantly affected Euclidean step placement in the perturbed step (all *P*<0.001). Also, for the perturbed step, there was a significant interaction of magnitude/direction, magnitude/timing, direction/timing and magnitude/direction/timing (all *P*<0.001). The ANOVA also revealed that magnitude, direction and timing all significantly affected Euclidean step placement in the recovery step (all *P*<0.001). Also, for the recovery step, there was a significant interaction of magnitude/direction, magnitude/timing, direction/timing and magnitude/direction/timing (all *P*<0.001). Higher magnitude perturbations tended to cause more severe recovery strategies to be used in both the perturbed and recovery steps. The earliest onset timing (double stance) caused the most severe recovery strategy in the perturbed step, but the latest timing (late single stance) caused the most severe strategies in the recovery step. For perturbation direction, anteroposterior perturbations caused relatively minor recovery strategies to be used in both the perturbed and recovery steps, while perturbations with a mediolateral component tended to cause larger strategies to be used, particularly in the recovery step. Additionally, diagonal perturbations tended to elicit similar responses to mediolateral perturbations and more severe than anteroposterior perturbations. Fig. S1 is a comparable figure that individually evaluates step length and width.

### Jump occurrences

Jumps occurred for both the 10 cm (4/27 jumps) and 15 cm (23/27 jumps) magnitudes, though only 0.40% of 10 cm perturbations elicited jumps (thus it is not visible in Fig. 4A,D) in comparison to 2.2% of 15 cm perturbations. Jumps primarily occurred in the late single stance onset time (18/27 jumps), with a small portion also occurring in the mid single stance (8/27 jumps) and double stance (1/27 jumps) onset times. Jumps primarily occurred during lateral perturbations (15/27 jumps), which caused the participant to fall toward the inside of their stance foot, and anterolateral perturbations (10/27 jumps), which caused the participant to fall toward the inside of their stance foot and backward. A small portion of the jumps were caused by medial (1/27 jumps) and anteromedial (1/27 jumps) perturbations. Table S4 reports the perturbation conditions that elicited a jump and the number of jumps that occurred for each of those conditions.

### Balance and recovery strategies

**Fig. 5.**
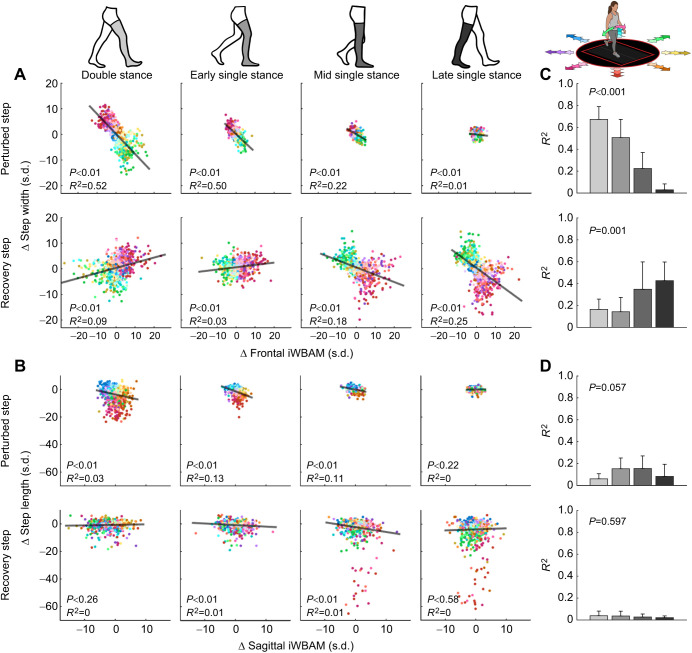
**Correlation between iWBAM deviation and step placement deviation for both the perturbed and recovery steps.** (A,B) Results from all participants are shown. The effect of frontal iWBAM on step width (A) and of sagittal iWBAM on step length (B) for each perturbation onset time. The hue of each data point indicates the perturbation direction, while the shade indicates the magnitude, shown in the walking arrow diagram in the upper right. The best fit line and the corresponding statistics are shown in each plot. (C,D) The correlation strength values for participant-specific correlations for frontal iWBAM and step width (C) and sagittal iWBAM and step length (D). Each bar corresponds to different perturbation onset times, aligned with the walking diagram at the top of the figure and the across-participant scatter plots. Note that correlation strengths shown in the bar charts are higher than in the scatter plots (A,B) because they show participant-specific correlations, whereas the scatter plots show data points from all participants. The results of one-way ANOVAs analyzing the effect of timing on the strength of the participant-specific correlations are shown in each plot.

We investigated whether changes in stability influenced changes in step placement by evaluating the relationship between iWBAM and step placement in the perturbed and recovery steps, shown broken out across perturbation onset times (Fig. 5A,B). There was a significant (*P*<0.05) relationship between frontal iWBAM and step width in both the perturbed and recovery steps across all perturbation onset times. However, the correlation strength between frontal iWBAM and step width varied depending on perturbation onset time. In the perturbed step, the correlation between frontal iWBAM and step width in double stance (*R*^2^=0.52) and early single stance (*R*^2^=0.5) steps was stronger than in mid single stance (*R*^2^=0.22) and late single stance (*R*^2^=0.01) steps. However, in the recovery step, this trend reversed, as the correlation between frontal iWBAM and step width in double stance (*R*^2^=0.09) and early single stance (*R*^2^=0.03) steps was weaker than in mid single stance (*R*^2^=0.18) and late single stance (*R*^2^=0.25) steps. There was a significant (*P*<0.05) relationship between sagittal iWBAM and step length in the perturbed steps for perturbation onset times in double stance, early single stance and mid single stance. Of these significant correlations, all of them were very weak (*R*^2^=0.03, 0.13 and 0.11 for double stance, early single stance and mid single stance, respectively). There was also a significant (*P*<0.05) relationship between sagittal iWBAM and step length in the recovery steps with perturbation onset times in early single stance and mid single stance. Both of these significant correlations were again extremely weak (*R*^2^=0.01 for both early single stance and mid single stance). All statistical values are also shown in the correlation plots in Fig. 5.

Next, we evaluated whether perturbation onset time had a significant effect on the correlation strength of iWBAM and step placement (Fig. 5C,D). One-way ANOVAs revealed that perturbation onset time significantly affected (*P*<0.05) the correlation strength between frontal iWBAM and step width in both the perturbed (*P*<0.001) and recovery (*P*=0.001) steps. However, perturbation onset time did not significantly affect the correlation strength between sagittal iWBAM and step length in either the perturbed (*P*=0.057) or recovery (*P*=0.597) steps. Mean *R*^2^ values for individual participant linear correlations (shown in Fig. 5C,D) were consistently higher than the across-participant linear correlations shown in the scatter plots.

## DISCUSSION

### Perturbation conditions and balance

In both the perturbed and recovery steps, higher perturbation magnitudes caused greater deviation in Euclidean iWBAM, which we used to represent change in balance relative to steady state. This is unsurprising and aligns with previous literature that has observed similar effects of perturbation magnitude on other stability measures, such as COM velocity (Hof et al., 2010; Vlutters et al., 2016). The perturbation onset time with the most time until the next heel contact (double stance) caused the greatest deviation in Euclidean iWBAM in the perturbed step. We expected this for the perturbed step, as the earliest perturbation onset time implies the greatest amount of time until the next heel contact, allowing instability to propagate prior to the ability to make large COP changes with step placement. We anticipated that this prolonged time prior to heel contact would allow for a more intentional and effective step placement on the perturbed step, consistent with previous work (Golyski et al., 2022), causing improved stability during the recovery step. We saw this anticipated outcome reflected in our data, where later perturbation onset times caused the greatest deviation in Euclidean iWBAM in the recovery step. We expect that this is because later onset times (mid or late single stance) allow for little to no volitional step placement in the perturbed step and leave the burden of correcting for instability to the recovery step (Hof et al., 2010; Vlutters et al., 2018). Lastly, perturbation directions that caused a mediolateral pitch of the COM were the most destabilizing in the perturbed step. These were also very destabilizing in the recovery step, as well as perturbations that contributed a simultaneous mediolateral and backward pitch of the COM.

### Perturbation conditions and recovery strategies

Similar to the balance responses, higher magnitude perturbations caused greater deviation in Euclidean step placement, which quantifies the total step placement deviation relative to steady state. This is consistent with previous literature that showed greater deviation in both step length and width with greater perturbation magnitude (Vlutters et al., 2016). The earliest (double stance) perturbation onset time caused the largest deviation in step placement in the perturbed step, while the latest perturbation onset time (late single stance) caused the largest deviation in step placement in the recovery step. This reversal between the ‘worst’ perturbation time for the perturbed and recovery steps is what we expected; previous research determined that about 0.28 s is needed for sufficient lateral step placement with lateral pushes (Hof et al., 2010). Though this required amount of time likely varies with the magnitude of corrective step that is needed, it suggests that participants are not able to achieve sufficient step placement in the perturbed step following mid and late single stance perturbations (approximately 0.2 and 0.1 s prior to heel contact, respectively, assuming a 1 s gait cycle). This would then shift the burden of corrective step placement to the recovery step for later perturbations. Lastly, perturbations in the lateral (COM pitch toward the inside of the stance foot, requiring wider step) and lateral–anterior (COM pitch backward and toward the inside of the stance foot) directions caused the greatest step placement changes in the perturbed step. However, both medial and lateral perturbations caused the highest step placement deviation in the recovery step. It is possible that the Euclidean step placement changes with mediolateral perturbations are driven by the necessity for step width changes; previous work by Vlutters et al. (2016) suggested that humans choose to use stance limb strategies for sagittal stabilization rather than step length changes because of the large energetic cost associated with altering sagittal step placement (Donelan et al., 2002). This would indicate that anteroposterior perturbations demanding sagittal COP adjustments do not induce large Euclidean step placement changes relative to mediolateral perturbations.

### Linking balance to recovery strategies

We hypothesized that frontal plane iWBAM would correlate with step width and sagittal plane iWBAM would correlate with step length during perturbed locomotion. There was a significant relationship between frontal iWBAM and step width across all perturbation onset times in both the perturbed and recovery steps, indicating that balance throughout a step influences the subsequent step width at the termination of the step. However, in the perturbed step, correlation strength went down for later onset times. This could reflect the step placement results discussed above, where later perturbation onset times may not provide sufficient time for volitional step width modulation, inhibiting the participant from responding to instability that begins later in the step.

In the recovery step, the correlation strength between frontal iWBAM and step width went up for later onset times, though the correlation strengths were weaker than those seen for the perturbed step for earlier perturbation onset times. The weaker correlations across onset times in the recovery step could be due to the participant having ample time to employ other strategies in combination with step placement, such as lateral ankle strategy (Hof et al., 2010) or using arm (Collins et al., 2009; Gholizadeh et al., 2019) or torso counter-rotation (Li and Fey, 2018) to induce momentum changes. However, this does not imply that those other strategies are not being used on the perturbed step; on the contrary, lateral ankle strategy is faster acting, as the participant does not have to wait until the subsequent heel contact to employ it (Reimann et al., 2017). The stronger correlations for the perturbed step may indicate that, although it is not the only recovery strategy being employed, participants relied more heavily on a stepping strategy in the perturbed step, specifically for earlier perturbation onset times.

The frontal plane data in Fig. 5 appear to fan out toward more extreme balance and step placement deviations. However, the underlying cause seems to be that different participants present a different sensitivity of step width in response to frontal iWBAM changes, causing shallower or steeper slopes of data points relative to the across-participant best fit line. Though an individual-participant scatter plot was not shown, this result is reflected in the *R*^2^ values in the bar plots in Fig. 5C, which show the correlation strength of the individual-participant linear correlations. This could be due to participants having different preferences for how quickly they correct for instability; two participants experiencing the same perturbation may not choose to execute the same step placement response.

Contrary to step width, these data provide little evidence that sagittal iWBAM similarly influences step length. Although there was still a significant relationship in both the perturbed and recovery steps, the correlations between sagittal iWBAM and step length were very weak in both the perturbed and recovery steps. Though the early and mid single stance onset times presented slightly stronger correlations on the perturbed step, we found no significant effect of timing on the correlation strength between sagittal iWBAM and step length in either step. The lack of correlation between sagittal iWBAM and step length reinforces previous work that determined, during sagittal plane perturbations, that humans predominantly used ankle moment modulation to correct for instability, limiting the need for step length modulation (Vlutters et al., 2016). Why then would the data presented in this work still show significant deviations in step length relative to steady state across both the perturbed and recovery steps? Interestingly, most of the severe step length deviations were perturbations with a mediolateral component (purely mediolateral perturbations shown with pink and green data points). This could be due to coupling between step length and width, in which step length would be altered when stabilization is required by step width modulation (Bauby and Kuo, 2000).

The results from this work indicate that individuals use stepping strategy to correct for instability following perturbations in the frontal plane but may be far less reliant on stepping strategy to correct for instability in the sagittal plane. In passive dynamic walking models where step placement is the only method for correcting instability, we would expect very strong correlations between balance measures and step placement (Hof, 2008). But, humans have the ability to correct for changes in WBAM using additional recovery strategies, including using the arms or torso to generate momentum and using ankle moments to shift the COP. Our results suggest that additional strategies accommodate stepping strategy, and that other strategies may be more dominant in correcting for sagittal plane instability. Future work could use a similar approach to investigate the correlation between arm momentum, torso momentum or ankle moments during stance to reveal how other strategies are employed to correct for instability across varying perturbation conditions.

Future work could also investigate how well these findings translate to other perturbation methodologies. The ground translation perturbation paradigm used here resembles some daily perturbations, such as walking down the aisle of a moving bus or an airplane experiencing turbulence, where the walking surface reference frame is moving. Other perturbations of interest may include slips, COM pulls or ground height changes that resemble other perturbations that people may encounter in their daily lives. To better address how findings from studies spanning these modalities translate to one another, future work could investigate how magnitude, direction, timing, additional perturbation characteristics and subsequent responses compare across perturbation types.

Broadly, this work contributes an understanding of how balance is affected by a diverse set of perturbation conditions and how individuals use stepping strategy to compensate for changes in balance. To accomplish this, we introduced a novel perturbation paradigm that is the first to vary perturbation magnitude, direction and timing in tandem, providing insight into a large variety of destabilizing scenarios. We found that both iWBAM and step placement are significantly influenced by magnitude, direction and timing, as well as by several interaction effects between variables. We also found that step width responses reflect changes in frontal iWBAM and are also influenced by the onset time of a perturbation, while step length responses show very little correlation with changes in sagittal iWBAM. Lastly, to expand the impact of this work, the comprehensive perturbation dataset is open-source which we hope will enable future work that investigates human balance and responses during perturbed locomotion.

### Limitations

Although we did our best to control the variables in this study, there are several limitations that may have affected our work. There was variability in the perturbation onset time, possibly caused by natural walking variability as well as mislabeling or missing markers during real-time tracking that informed the closed-loop timing controller for the perturbation platform. Additionally, repetitions for a given perturbation condition occurred on a randomized foot (right or left), but previous literature suggests that participants' motor control responses to instability may be modestly affected by leg dominance (Martelli et al., 2013), which we did not consider in our analysis. Lastly, participants' multi-step recovery strategies may be constrained by the use of a treadmill; thus, we constrained the analysis to the perturbed step and the immediate step following (recovery step). The bounds of the treadmill may result in stepping behavior that would not match overground perturbations or those that could be performed with a larger treadmill walking surface. Additionally, the treadmill remained at a fixed walking speed throughout the duration of the experiment, though individuals may transiently alter their walking speed in response to perturbations in unconstrained environments, which they only had a limited ability to do here without exiting the bounds of the treadmill.

## Supplementary Material

10.1242/jexbio.244760_sup1 Supplementary information

## References

[JEB244760C1] Afschrift, M., van Deursen, R., De Groote, F. and Jonkers, I. (2019). Increased use of stepping strategy in response to medio-lateral perturbations in the elderly relates to altered reactive tibialis anterior activity. *Gait Posture* 68, 575-582. 10.1016/j.gaitpost.2019.01.010 30654320

[JEB244760C2] Bauby, C. E. and Kuo, A. D. (2000). Active control of lateral balance in human walking. *J. Biomech.* 33, 1433-1440. 10.1016/S0021-9290(00)00101-9 10940402

[JEB244760C3] Berger, W., Dietz, V. and Quintern, J. (1984). Corrective reactions to stumbling in man: neuronal co-ordination of bilateral leg muscle activity during gait. *J. Physiol.* 357, 109-125. 10.1113/jphysiol.1984.sp015492 6512687 PMC1193250

[JEB244760C4] Bruijn, S. M., Meijer, O. G., Beek, P. J. and van Dieën, J. H. (2013). Assessing the stability of human locomotion: a review of current measures. *J. R. Soc. Interface.* 10, 20120999. 10.1098/rsif.2012.0999 23516062 PMC3645408

[JEB244760C5] Camargo, J., Ramanathan, A., Csomay-Shanklin, N. and Young, A. (2020). Automated gap-filling for marker-based biomechanical motion capture data. *Comput. Methods Biomech. Biomed. Engin.* 23, 1180-1189. 10.1080/10255842.2020.1789971 32654510

[JEB244760C6] Collins, S. H., Adamczyk, P. G. and Kuo, A. D. (2009). Dynamic arm swinging in human walking. *Proc. R. Soc. B* 276, 3679-3688. 10.1098/rspb.2009.0664 PMC2817299 19640879

[JEB244760C7] Delp, S. L., Anderson, F. C., Arnold, A. S., Loan, P., Habib, A., John, C. T., Guendelman, E. and Thelen, D. G. (2007). OpenSim: open-source software to create and analyze dynamic simulations of movement. *IEEE Trans. Biomed. Eng.* 54, 1940-1950. 10.1109/TBME.2007.901024 18018689

[JEB244760C8] Donelan, J. M., Kram, R. and Kuo, A. D. (2002). Mechanical work for step-to-step transitions is a major determinant of the metabolic cost of human walking. *J. Exp. Biol.* 205, 3717-3727. 10.1242/jeb.205.23.3717 12409498

[JEB244760C9] Eveld, M. E., King, S. T., Vailati, L. G., Zelik, K. E. and Goldfarb, M. (2021). On the basis for stumble recovery strategy selection in healthy adults. *J. Biomech. Eng.* 143, 071003. 10.1115/1.4050171 33590838 PMC8086400

[JEB244760C10] Gholizadeh, H., Hill, A. and Nantel, J. (2019). Effect of arm motion on postural stability when recovering from a slip perturbation | Elsevier Enhanced Reader. *J. Biomech.* 95, 109269. 10.1016/j.jbiomech.2019.07.013 31443945

[JEB244760C11] Golyski, P. R., Vazquez, E., Leestma, J. K. and Sawicki, G. S. (2022). Onset timing of treadmill belt perturbations influences stability during walking. *J. Biomech.* 130, 110800. 10.1016/j.jbiomech.2021.110800 34864443

[JEB244760C12] Herr, H. and Popovic, M. (2008). Angular momentum in human walking. *J. Exp. Biol.* 211, 467-481. 10.1242/jeb.008573 18245623

[JEB244760C13] Hof, A. L. (2008). The ‘extrapolated center of mass’ concept suggests a simple control of balance in walking. *Hum. Mov. Sci.* 27, 112-125. 10.1016/j.humov.2007.08.003 17935808

[JEB244760C14] Hof, A. L., Gazendam, M. G. J. and Sinke, W. E. (2005). The condition for dynamic stability. *J. Biomech.* 38, 1-8. 10.1016/j.jbiomech.2004.03.025 15519333

[JEB244760C15] Hof, A. L., Vermerris, S. M. and Gjaltema, W. A. (2010). Balance responses to lateral perturbations in human treadmill walking. *J. Exp. Biol.* 213, 2655-2664. 10.1242/jeb.042572 20639427

[JEB244760C16] Horak, F. B. and Nashner, L. M. (1986). Central programming of postural movements: adaptation to altered support-surface configurations. *J. Neurophysiol.* 55, 1369-1381. 10.1152/jn.1986.55.6.1369 3734861

[JEB244760C17] Joshi, V. and Srinivasan, M. (2019). A controller for walking derived from how humans recover from perturbations. *J. R. Soc. Interface.* 16, 20190027. 10.1098/rsif.2019.0027 31409232 PMC6731497

[JEB244760C18] Kazanski, M. E., Cusumano, J. P. and Dingwell, J. B. (2020). How healthy older adults regulate lateral foot placement while walking in laterally destabilizing environments. *J. Biomech.* 104, 109714. 10.1016/j.jbiomech.2020.109714 32139095 PMC7188576

[JEB244760C19] Li, W. and Fey, N. P. (2018). Neuromechanical Control Strategies of Frontal-Plane Angular Momentum of Human Upper Body During Locomotor Transitions. In 2018 7th IEEE International Conference on Biomedical Robotics and Biomechatronics (Biorob), pp. 984-989.

[JEB244760C20] Li, J. and Huang, H. J. (2022). Small directional treadmill perturbations induce differential gait stability adaptation. *J. Neurophysiol.* 127, 38-55. 10.1152/jn.00091.2021 34851745 PMC8721900

[JEB244760C21] Liu, C., Macedo, L. D. and Finley, J. M. (2018). Conservation of reactive stabilization strategies in the presence of step length asymmetries during walking. *Front. Hum. Neurosci.* 12, 251. 10.3389/fnhum.2018.00251 29997488 PMC6030543

[JEB244760C22] Major, M. J., Serba, C. K. and Gordon, K. E. (2020). Perturbation recovery during walking is impacted by knowledge of perturbation timing in below-knee prosthesis users and non-impaired participants. *PLoS ONE* 15, e0235686. 10.1371/journal.pone.0235686 32658907 PMC7357748

[JEB244760C23] Martelli, D., Monaco, V., Luciani, L. B. and Micera, S. (2013). Angular momentum during unexpected multidirectional perturbations delivered while walking. *IEEE Trans. Biomed. Eng.* 60, 1785-1795. 10.1109/TBME.2013.2241434 23358944

[JEB244760C24] Nolasco, L. A., Silverman, A. K. and Gates, D. H. (2019). Whole-body and segment angular momentum during 90-degree turns. *Gait Posture* 70, 12-19. 10.1016/j.gaitpost.2019.02.003 30776765

[JEB244760C25] Nott, C. R., Neptune, R. R. and Kautz, S. A. (2014). Relationships between frontal-plane angular momentum and clinical balance measures during post-stroke hemiparetic walking. *Gait Posture* 39, 129-134. 10.1016/j.gaitpost.2013.06.008 23820449 PMC3823741

[JEB244760C26] Popovic, M., Hofmann, A. and Herr, H. (2004). Angular momentum regulation during human walking: biomechanics and control. In IEEE International Conference on Robotics and Automation, 2004. Proceedings. ICRA ‘04. 2004, pp. 2405-2411. Vol. 3.

[JEB244760C27] Rajagopal, A., Dembia, C. L., DeMers, M. S., Delp, D. D., Hicks, J. L. and Delp, S. L. (2016). Full-body musculoskeletal model for muscle-driven simulation of human gait. *IEEE Trans. Biomed. Eng.* 63, 2068-2079. 10.1109/TBME.2016.2586891 27392337 PMC5507211

[JEB244760C28] Reimann, H., Fettrow, T. D., Thompson, E. D., Agada, P., McFadyen, B. J. and Jeka, J. J. (2017). Complementary mechanisms for upright balance during walking. *PLoS ONE* 12, e0172215. 10.1371/journal.pone.0172215 28234936 PMC5325219

[JEB244760C29] Seth, A., Hicks, J. L., Uchida, T. K., Habib, A., Dembia, C. L., Dunne, J. J., Ong, C. F., DeMers, M. S., Rajagopal, A., Millard, M. et al. (2018). OpenSim: Simulating musculoskeletal dynamics and neuromuscular control to study human and animal movement. *PLoS Comput. Biol.* 14, e1006223. 10.1371/journal.pcbi.1006223 30048444 PMC6061994

[JEB244760C30] Silverman, A. K. and Neptune, R. R. (2011). Differences in whole-body angular momentum between below-knee amputees and non-amputees across walking speeds. *J. Biomech.* 44, 379-385. 10.1016/j.jbiomech.2010.10.027 21074161

[JEB244760C31] Silverman, A. K., Wilken, J. M., Sinitski, E. H. and Neptune, R. R. (2012). Whole-body angular momentum in incline and decline walking. *J. Biomech.* 45, 965-971. 10.1016/j.jbiomech.2012.01.012 22325978

[JEB244760C32] Tan, G. R., Raitor, M. and Collins, S. H. (2020). Bump'em: an Open-Source, Bump-Emulation System for Studying Human Balance and Gait. In 2020 IEEE International Conference on Robotics and Automation (ICRA), pp. 9093-9099. Paris, France: IEEE.

[JEB244760C33] Vlutters, M., Van Asseldonk, E. H. F. and Van der Kooij, H. (2016). Center of mass velocity based predictions in balance recovery following pelvis perturbations during human walking. *J. Exp. Biol.* 219, 1514-1523. 10.1242/jeb.129338 26994171

[JEB244760C34] Vlutters, M., Van Asseldonk, E. H. F. and van der Kooij, H. (2018). Foot placement modulation diminishes for perturbations near foot contact. *Front. Bioeng. Biotechnol.* 6, 48. 10.3389/fbioe.2018.00048 29868570 PMC5953331

[JEB244760C35] Wang, Y. and Srinivasan, M. (2014). Stepping in the direction of the fall: the next foot placement can be predicted from current upper body state in steady-state walking. *Biol. Lett.* 10, 20140405. 10.1098/rsbl.2014.0405 25252834 PMC4190959

[JEB244760C36] Zeni, J. A. and Higginson, J. S. (2010). Gait parameters and stride-to-stride variability during familiarization to walking on a split-belt treadmill. *Clin. Biomech.* 25, 383-386. 10.1016/j.clinbiomech.2009.11.002 PMC2847055 20004501

[JEB244760C37] Zeni, J. A., Richards, J. G. and Higginson, J. S. (2008). Two simple methods for determining gait events during treadmill and overground walking using kinematic data. *Gait Posture* 27, 710-714. 10.1016/j.gaitpost.2007.07.007 17723303 PMC2384115

