# Supplementary material for: Linking whole-body angular momentum and step placement during perturbed human walking
Source: J Exp Biol. 2023 Mar 29;226(6):jeb244760. doi: 10.1242/jeb.244760 (PMC10112983; doi:10.1242/jeb.244760)
Supplement: Supplementary information [file jexbio-226-244760-s1.pdf]

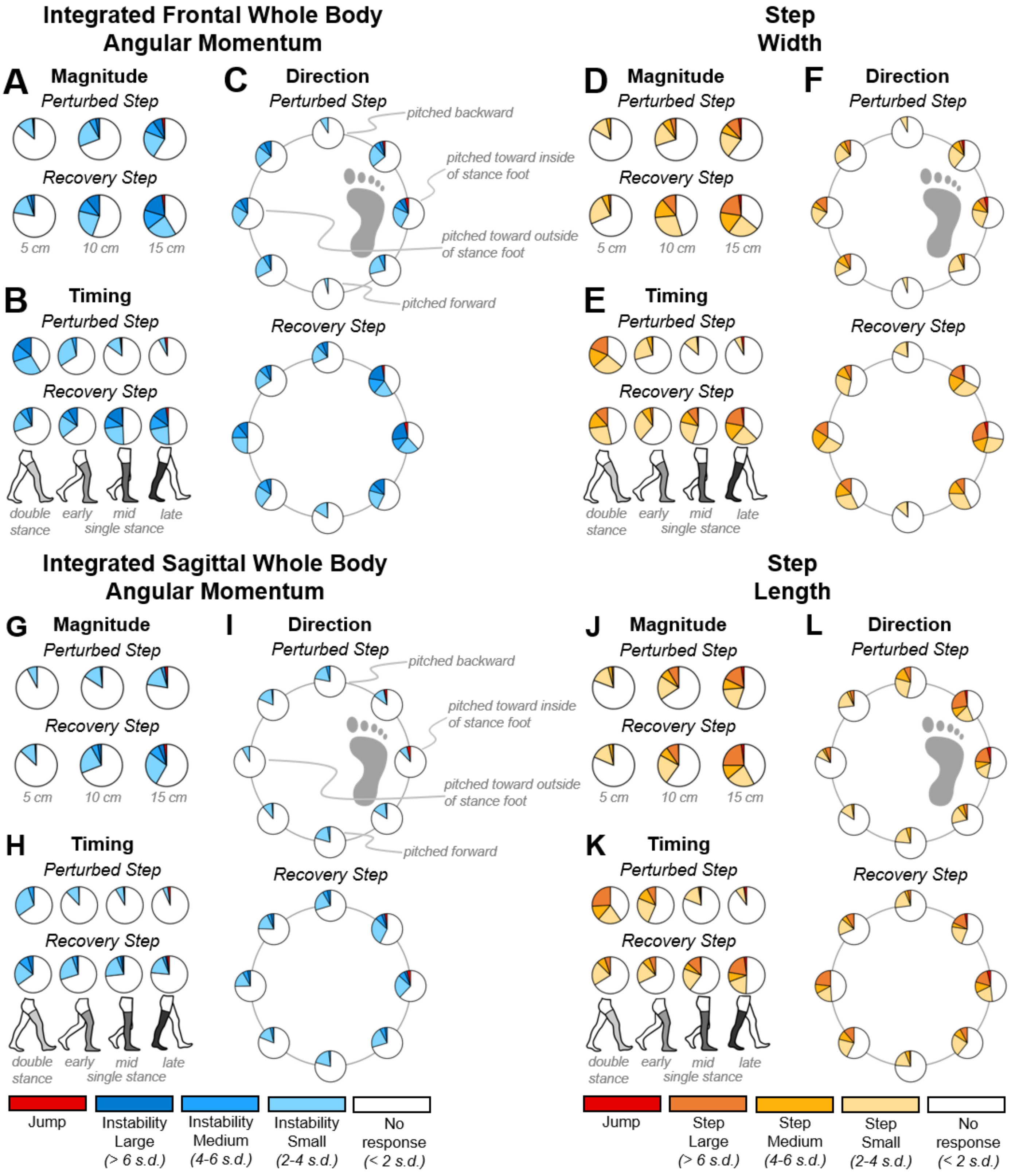

Fig. S1. Frontal and sagittal iWBAM and step width and length changes across perturbation conditions. Results from all participants are shown in the figure. **(A-C)** Deviations in frontal balance, measured by changes in integrated whole-body angular momentum (iWBAM) relative to steady state, across perturbation conditions for the perturbed and recovery steps. Severity of deviation is classified as no response as well as small, medium, and large changes in stability. (D-F) Deviations in step width across perturbation conditions for the perturbed and recovery steps. Severity of deviation is classified as no response as well as small, medium, and large changes in step placement. **(G-I)** Deviations in sagittal balance, measured by changes in iWBAM relative to steady state, across perturbation conditions for the perturbed and recovery steps. Severity of deviation is classified as no response as well as small, medium, and large changes in stability. **(J-L)** Deviations in step length across perturbation conditions for the perturbed and recovery steps. Severity of deviation is classified as no response as well as small, medium, and large changes in step placement. (ALL) An additional class, jump, is shown for trials in which the participant lost ground contact. If a jump occurred following the perturbation, the jump classification was shown for both the perturbed and recovery step.

Journal of Experimental Biology • Supplementary information

Table S1. iWBAM and step placement across perturbation magnitudes. Results from all participants are shown in the table. The results reported here are the numerical values associate with Fig. 4A,D in the main text, rounded to the nearest tenth decimal place.

Effect of Perturbation Magnitude on Euclidean iWBAM						
**Step**	**Magnitude** (cm)	**Jump (%)**	**Large (%)**	**Medium (%)**	**Small (%)**	**No Response (%)**
**Perturbed**	**5**	0	0.8	1.3	23.4	74.5
	**10**	0.4	3.1	8.1	34.1	54.3
	**15**	2.2	8.7	13.0	31.9	44.1
**Recovery**	**5**	0	3.2	5.1	29.9	61.7
	**10**	0.4	13.9	15.3	35.1	35.3
	**15**	2.2	24.3	22.5	29.3	21.7
**Effect of Perturbation Magnitude on Step Placement**						
**Perturbed**	**5**	0	2.3	5.9	27.8	64.1
	**10**	0.4	13.5	10.9	29.2	46.0
	**15**	2.2	22.4	11.1	28.2	36.2
**Recovery**	**5**	0	4.0	8.9	35.9	51.1
	**10**	0.4	21.3	18.2	31.9	28.3
	**15**	2.2	38.1	17.1	23.4	19.2

Table S2. iWBAM and step placement across perturbation directions. Results from all participants are shown in the table. The results reported here are the numerical values associate with Fig. 4C,F in the main text, rounded to the nearest tenth decimal place.

Step	Direction	Jump (%)	Large (%)	Medium (%)	Small (%)	No Response (%)
**Effect of Perturbation Direction on Euclidean iWBAM**						
**Perturbed**	**lateral**	3.8	6.3	8.8	31.3	49.7
	**lateral/anterior**	2.5	3.8	7.3	32.0	54.4
	**anterior**	0	0.5	5.1	26.5	67.9
	**medial/anterior**	0.3	8.4	8.9	30.7	51.8
	**medial**	0.3	8.1	9.8	30.3	51.5
	**medial/posterior**	0	4.3	8.0	31.2	56.5
	**posterior**	0	0	2.8	26.1	71.1
	**lateral/posterior**	0	2.3	9.3	30.3	58.3
**Recovery**	**lateral**	3.8	28.0	16.2	29.8	22.2
	**lateral/anterior**	2.5	26.2	20.9	29.2	21.2
	**anterior**	0	9.7	13.0	33.9	43.4
	**medial/anterior**	0.3	8.4	11.9	29.9	49.5
	**medial**	0.3	15.7	17.2	33.3	33.6
	**medial/posterior**	0	6.3	16.8	31.7	45.2
	**posterior**	0	0.3	5.3	32.0	62.4
	**lateral/posterior**	0	16.0	13.3	31.8	39.0
**Effect of Perturbation Direction on Step Placement**						
**Perturbed**	**lateral**	3.8	23.5	10.9	22.0	39.9
	**lateral/anterior**	2.5	28.2	10.1	23.2	36.0
	**anterior**	0	9.2	13.3	31.6	45.9
	**medial/anterior**	0.3	9.1	8.4	36.3	45.9
	**medial**	0.3	15.2	5.8	27.3	51.5
	**medial/posterior**	0	8.0	10.8	28.6	52.5
	**posterior**	0	0.3	5.1	28.9	65.7
	**lateral/posterior**	0	8.5	10.0	29.3	52.3
**Recovery**	**lateral**	3.8	37.6	15.2	28.5	14.9
	**lateral/anterior**	2.5	28.7	18.9	30.7	19.1
	**anterior**	0	3.6	6.6	34.2	55.6
	**medial/anterior**	0.3	15.2	15.7	30.5	38.3
	**medial**	0.3	36.1	19.2	23.5	21.0
	**medial/posterior**	0	26.4	17.8	28.4	27.4
	**posterior**	0	2.5	7.6	27.7	62.2
	**lateral/posterior**	0	18.8	16.8	39.8	24.8

Table S3. iWBAM and step placement across perturbation timings. Results from all participants are shown in the table. The results reported here are the numerical values associate with Fig. 4B,E in the main text, rounded to the nearest tenth decimal place.

Step	Timing	Jump (%)	Large (%)	Medium (%)	Small (%)	No Response (%)
**Effect of Perturbation Timing on Euclidean iWBAM**						
**Perturbed**	**DS**	0.1	16.4	22.3	40.6	20.7
	**early SS**	0	0.4	6.0	40.9	52.7
	**mid SS**	1.0	0	1.7	24.6	72.7
	**late SS**	2.2	0	0.2	14.3	83.3
**Recovery**	**DS**	0.1	10.8	13.7	31.5	43.8
	**early SS**	0	11.3	13.5	31.7	43.4
	**mid SS**	1.0	18.0	14.1	32.8	34.1
	**late SS**	2.2	15.0	15.8	29.9	37.1
**Effect of Perturbation Timing on Step Placement**						
**Perturbed**	**DS**	0.1	40.7	19.3	24.2	15.7
	**early SS**	0	10.2	15.3	40.1	34.4
	**mid SS**	1.0	0.4	2.9	32.3	63.5
	**late SS**	2.2	0.1	0.4	17.8	79.5
**Recovery**	**DS**	0.1	19.0	16.2	35.5	29.1
	**early SS**	0	10.5	12.7	34.8	42.0
	**mid SS**	1.0	20.3	13.0	29.9	35.7
	**late SS**	2.2	33.9	16.8	21.9	25.2

Table S4. All conditions that elicited a jump response and the number of jump occurrences. Results from all participants are shown in the table.

**Magnitude** (cm)	**Direction**	**Timing**	**Jump Occurrences**
15	lateral (pink)	late single stance	8
15	antero-lateral (red)	late single stance	5
15	antero-lateral (red)	mid single stance	4
15	lateral (pink)	mid single stance	4
10	lateral (pink)	late single stance	3
10	antero-lateral (red)	late single stance	1
15	antero-medial (yellow)	late single stance	1
15	medial (green)	double stance	1

Journal of Experimental Biology • Supplementary information
